# Decrease of CD68 Synovial Macrophages in Celastrol Treated Arthritic Rats

**DOI:** 10.1371/journal.pone.0142448

**Published:** 2015-12-11

**Authors:** Rita Cascão, Bruno Vidal, Inês P. Lopes, Eunice Paisana, José Rino, Luis F. Moita, João E. Fonseca

**Affiliations:** 1 Instituto de Medicina Molecular, Faculdade de Medicina da Universidade de Lisboa, Lisbon, Portugal; 2 Instituto Gulbenkian de Ciência, Oeiras, Portugal; 3 Rheumatology Department, Centro Hospitalar de Lisboa Norte, EPE, Hospital de Santa Maria, Lisbon Academic Medical Centre, Lisbon, Portugal; Singapore Immunology Network, Agency for Science, Technology and Research (A*STAR), SINGAPORE

## Abstract

**Background:**

Rheumatoid arthritis (RA) is a chronic immune-mediated inflammatory disease characterized by cellular infiltration into the joints, hyperproliferation of synovial cells and bone damage. Available treatments for RA only induce remission in around 30% of the patients, have important adverse effects and its use is limited by their high cost. Therefore, compounds that can control arthritis, with an acceptable safety profile and low production costs are still an unmet need. We have shown, *in vitro*, that celastrol inhibits both IL-1β and TNF, which play an important role in RA, and, *in vivo*, that celastrol has significant anti-inflammatory properties. Our main goal in this work was to test the effect of celastrol in the number of sublining CD68 macrophages (a biomarker of therapeutic response for novel RA treatments) and on the overall synovial tissue cellularity and joint structure in the adjuvant-induced rat model of arthritis (AIA).

**Methods:**

Celastrol was administered to AIA rats both in the early (4 days after disease induction) and late (11 days after disease induction) phases of arthritis development. The inflammatory score, ankle perimeter and body weight were evaluated during treatment period. Rats were sacrificed after 22 days of disease progression and blood, internal organs and paw samples were collected for toxicological blood parameters and serum proinflammatory cytokine quantification, as well as histopathological and immunohistochemical evaluation, respectively.

**Results:**

Here we report that celastrol significantly decreases the number of sublining CD68 macrophages and the overall synovial inflammatory cellularity, and halted joint destruction without side effects.

**Conclusions:**

Our results validate celastrol as a promising compound for the treatment of arthritis.

## Introduction

Rheumatoid arthritis (RA) is a chronic immune mediated inflammatory disease that is mainly characterized by hyperproliferation of synovial cells, infiltration of mononuclear cells into the synovium and early destruction of articular cartilage and bone, causing progressive damage to the musculoskeletal system and consequently the loss of physical function and life quality [1–3]. The most debilitating feature of RA is joint destruction, which is derived from an uncontrolled inflammatory process. RA joint synovial cellular infiltrate consists of activated macrophages, B and T cells, which secrete proinflammatory cytokines and other mediators of inflammation [1, 4, 5] that not only perpetuate the inflammatory process but also increase bone resorption [6–10]. In addition, activated synovial fibroblasts, chondrocytes and osteoclasts contribute to the underlying cartilage and bone damage [11]. Despite this clear link between inflammation and increased bone turnover in RA and the existence of several therapeutical options, their efficacy on inflammation and bone treatment seem to be uncoupled, with some drugs suppressing inflammation but failing to protect bone [12, 13] and others halting bone destruction but with no effect on controlling inflammation [14]. Moreover, drugs used to treat RA, ranging from nonsteroidal anti-inflammatory drugs (NSAIDs) to disease-modifying antirheumatic drugs (DMARDs), and biological DMARDs, still cause severe side effects [15, 16] and are only able to induce remission in around 20–30% of the patients, leaving the majority of the individuals affected by RA with a chronic inflammatory process that will lead to damage. In addition to this, the most recent and innovative treatments are highly expensive, representing a burden to national health services and creating a barrier to its use in less effluent areas of the world. Therefore, compounds that can control arthritis, with an acceptable safety profile and low production cost are still an unmet need.

In this context, we have recently identified celastrol, a pentacyclic triterpenoid compound isolated from the roots of the Chinese herb Tripterygium wilfordii Hook F, as a potential RA therapeutic candidate [17]. We have shown that celastrol inhibits both interleukin (IL)-1β and tumour necrosis factor (TNF), which play an important role since the early phase of RA [18], and has significant anti-inflammatory and anti-proliferative properties in an adjuvant-induced rat model of arthritis (AIA) [17]. Supporting our own results, other studies using celastrol have reported beneficial effects in various models of inflammation, diminishing joint swelling and damage, serum IgG level, TNF and IL-1β mRNA and preventing disease progression [19]. Importantly, recent studies have also demonstrated that celastrol protects human chondrocytes by down-regulating the expression of metalloproteinases (MMPs) and inducible nitric oxide synthase (iNOS), suppresses several chemokines that mediate cellular joint infiltration [20], impairs B cell development [21] and also regulates bone remodelling-related immune mediators and proinflammatory cytokines in AIA synovium-infiltrating cells cultured *ex vivo* and in the RAW264.7 macrophagic cell line [22]. Celastrol might thus constitute an attractive candidate to have an early effect not only in controlling inflammation but also in preventing bone structural disturbances that occur in arthritis.

The efficacy of new compounds in the treatment of RA has been associated with a decrease in CD68 positive macrophages in the synovial sublining layer. This effect has been clearly demonstrated for most of the effective treatments for RA, including classic treatments, such as prednisolone [23], gold salts [24], methotrexate [25, 26] and leflunomide [27], and also for biologics such as infliximab [28, 29], anakinra [30, 31] and rituximab [32]. Interestingly, a study of a CCL-2/MCP-1 monoclonal antibody antagonist demonstrated no change in CD68 sublining macrophages and this was associated with no change in disease activity [33]. In accordance, a C5aR antagonist did not affect CD68 sublining macrophages and no clinical effect occurred [34]. Furthermore, a multicenter study on the correlation of the number of sublining CD68 cells and the change in DAS28 demonstrated excellent inter-centre agreement [32] and it has been shown that the number of CD68 macrophages decreases with a reduction in disease activity as measured by Disease Activity Score [35]. Due to these very solid evidences, the number of CD68 sublining macrophages has been proposed as a biomarker of therapeutic response to be used in the test of novel treatments for RA [32]. Of interest, in the preclinical test of new compounds, a number of observations have shown that effective RA treatments such as tofacitinib [36] and methotrexate [37] also decrease CD68 sublining macrophages in animal models of arthritis. Several experimental compounds have also shown an association between control of arthritis and reduction in the number of CD68 macrophages in animal models of arthritis [38–40].

Our aim in the herein study was to test the effect of celastrol treatment in the number of sublining CD68 macrophages and on the overall synovial tissue cellularity and joint structure in an animal model of arthritis, as a further argument to its possible efficacy in RA treatment.

In this work we report that celastrol significantly decreases the number of sublining CD68 macrophages and the overall synovial inflammatory cellularity, and halted joint destruction without any detectable side effects.

## Materials and Methods

### Animal experimental design

Eight-week-old female wistar AIA rats were purchased from Charles River Laboratories International (Massachusetts, USA). AIA rats were maintained under specific pathogen free (SPF) conditions and housed per groups under standard laboratory conditions (at 22°C under 12-hour light/12-hour dark conditions). Humane end-points were established and animals were sacrificed when presenting the maximum inflammatory score in more than 2 paws or when presenting more than 20% of body weight loss. All experiments were approved by the Animal User and Ethical Committees at the Instituto de Medicina Molecular (Lisbon University), according to the Portuguese law and the European recommendations. The dose of celastrol (1μg/g body weight daily) used in this study was based on that used in our previous study [17] and in other studies [22]. The need for daily administrations is also supported by Zhang J. et al who showed that the half-life of pure celastrol is approximately 10 hours [41]. Celastrol (Sigma, Missouri, USA) stock solution of 100mg/ml in DMSO was dissolved in normal saline solution and injected intraperitoneally in AIA rats after 4 days (early treatment group) and after 11 days (late treatment group) of disease induction, when arthritis was already present. A group of healthy non-arthritic and arthritic untreated female age-matched wistar rats sacrificed at day 4 (baseline for the celastrol early-treated group, at preclinical stage, N = 13), day 11 (baseline for the celastrol late-treated group, at acute clinical stage, N = 18) and day 22 after disease induction (chronic clinical stage) were used as controls in all experiments for comparison. At the preclinical AIA progression stage evidence of inflammation or bone erosion is still lacking in the contralateral hind paw and fore paws. Hind paw swelling, inflammation and joint erosions are steadily progressing during acute clinical stage and reach a plateau in the chronic stage [42]. The inflammatory score, ankle perimeter and body weight were measured during the period of treatment. Inflammatory score was evaluated by counting the score of each paw joint in a scale of 0–3 (0—absence; 1—erythema; 2—erythema and swelling; 3—deformities and functional impairment). The total score of each animal was defined as the sum of the partial scores of each affected joint [17, 43]. Rats were sacrificed by CO_2_ narcosis and blood, internal organs as well as paw samples were collected.

### Toxicological evaluation

For histopathological observation, lung, liver, kidney and spleen samples were collected at the time of sacrifice. Samples were fixed immediately in 10% neutral buffered formalin solution and then dehydrated with increasing ethanol concentrations (70%, 96% and 100%). Samples were next embedded in paraffin, sectioned using a microtome, mounted on microscope slides and stained with hematoxylin and eosin. Tissue histopathological changes were examined by a pathologist blinded to the experimental groups. All images were acquired using a Leica DM 2500 microscope equipped with a color camera Leica MC170 HD (Leica microsystems, Wetzlar, Germany). Moreover, blood toxicological parameters, such as creatine kinase, urea, lactate dehydrogenase and alanine transaminase, were measured in serum samples by enzyme linked immunosorbent assay (ELISA) technique according to the manufacturer’s instructions (BioAssay Systems, California, USA). Samples were analyzed using a plate reader Infinite M200 (Tecan, Mannedorf, Switzerland).

### Systemic cytokine quantification

Proinflammatory cytokines IL-1β (Boster Bio, California, USA), IL-6 (Boster Bio, California, USA), IL-17 (Sunred Biological Technology, Shanghai, China) and TNF (RayBiotech, Georgia, USA) were quantified in serum samples using specific rat ELISA kits according to the provider's recommendations. Standard curves for each cytokine were generated by using reference cytokine concentrations supplied by the manufacturer. Samples were analyzed using a plate reader Infinite M200 (Tecan, Mannedorf, Switzerland).

### Histological and immunohistochemical evaluation of hind paws

Left hind paw samples collected at the time of sacrifice were fixed immediately in 10% neutral buffered formalin solution and then decalcified in 10% formic acid. Samples were next dehydrated and embedded in paraffin, serially sectioned at a thickness of 5 μm using a microtome, mounted on microscope slides and stained with hematoxylin and eosin for morphological examination of structural changes and cellular infiltration. Histopathological evaluation of rat joints was performed in a blind fashion using 4 semi-quantitative scores: Sublining layer infiltration score (0—none to diffuse infiltration; 1—lymphoid cell aggregate; 2—lymphoid follicles; 3—lymphoid follicles with germinal center formation); Lining layer cell number score (0—fewer than three layers; 1—three to four layers; 2—five to six layers; 3—more than six layers); Bone erosion score (0—no erosions; 1—minimal; 2—mild; 3—moderate; 4—marked); Global severity score (0—no signs of inflammation; 1—mild; 2—moderate; 3—severe) [17, 44, 45]. Paw sections were also used for immunohistochemical staining with CD68 (Abcam, Cambridge, UK), CD163 (Biorbyt, Massachusetts, USA), CD3 (Abcam, Cambridge, UK), CD19 (Biorbyt, Massachusetts, USA) and Ki67 (Abcam, Cambridge, UK) antibodies. Tissue sections were incubated with the primary antibody and with EnVision+ (Dako, Glostrup, Denmark). Color was developed in solution containing diaminobenzadine-tetrahydrochloride (Sigma, Missouri, USA), 0.5% H_2_O_2_ in phosphate-buffered saline buffer (pH 7.6). Slides were counterstained with hematoxylin and mounted. Immunohistochemical evaluation of rat joints was performed in a blind fashion using a semi-quantitative score of 0–4 (0—no staining; 1–0–25% staining; 2–25–50% staining; 3–50–75% staining; 4—more than 75% staining) [17]. Images were acquired using a Leica DM2500 (Leica Microsystems, Wetzlar, Germany) microscope equipped with a color camera.

For a quantitative analysis of the immunohistochemical staining, we acquired whole-slide color images of single tissue slides using a NanoZoomer SQ slide scanner (Hamamatsu Photonics, Hamamatsu City, Japan) with 20x magnification (0.46 μm resolution). We developed an image analysis software written in MATLAB (Mathworks, Natick, MA) to identify and count the number of positive cells that displayed a specific cytoplasmic staining in representative sections. Briefly, single cell nuclei stained with hematoxylin were identified by color thresholding in the L*a*b* color space with the range of parameters L* = [40,72], a* = [–11,20] and b* = [–37,12] followed by particle analysis. Dilated regions of interest (ROIs) with a radius of 5 pixels were next defined for each detected particle as the cytoplasmic area. The antibody staining was also identified by color thresholding in the L*a*b* color space with the range of parameters L* = [40,80], a* = [–6,20] and b* = [-0.2,33]. Each cell ROI was then evaluated for antibody positive staining, defined by the occurrence of at least 20 pixels with a color value included in the cytoplasmic L*a*b* threshold range. We cropped areas of interest from whole-slide color images corresponding to synovial membranes and the software was set to batch process all images and output the total number of cells and the number of cells with positive antibody staining for each section. Then the density of positive cells was calculated by dividing the positive cell count by the area value.

### Statistical analysis

Statistical differences were determined with non-parametric Kruskal-Wallis (Dunn´s Multiple Comparison tests) and Mann–Whitney tests using GraphPad Prism (GraphPad, California, USA). Correlation analysis was performed with the Spearman test. Differences were considered statistically significant for p<0.05.

## Results

### Celastrol safely suppresses inflammatory manifestations in rat adjuvant-induced arthritis

To further validate the *in vivo* anti-inflammatory effect of celastrol in the context of arthritis, we have used the AIA rat model. The AIA experimental arthritis shares some characteristics of RA, such as hyperplasia of the synovial membrane, inflammatory infiltration of the joints, deposition of immune complexes in articular cartilage, pannus formation and destruction of bone. This model is also useful to characterize treatment responses by the reduction of inflammation or changes in the synovial tissue [46]. Overall, the AIA model has been extensively used to clarify the mechanisms of human RA pathogenesis and to identify potential targets and new drugs for therapeutic intervention [47], and has thus been our model of choice for our first experimental use of celastrol [17, 48].

**Fig 1:**
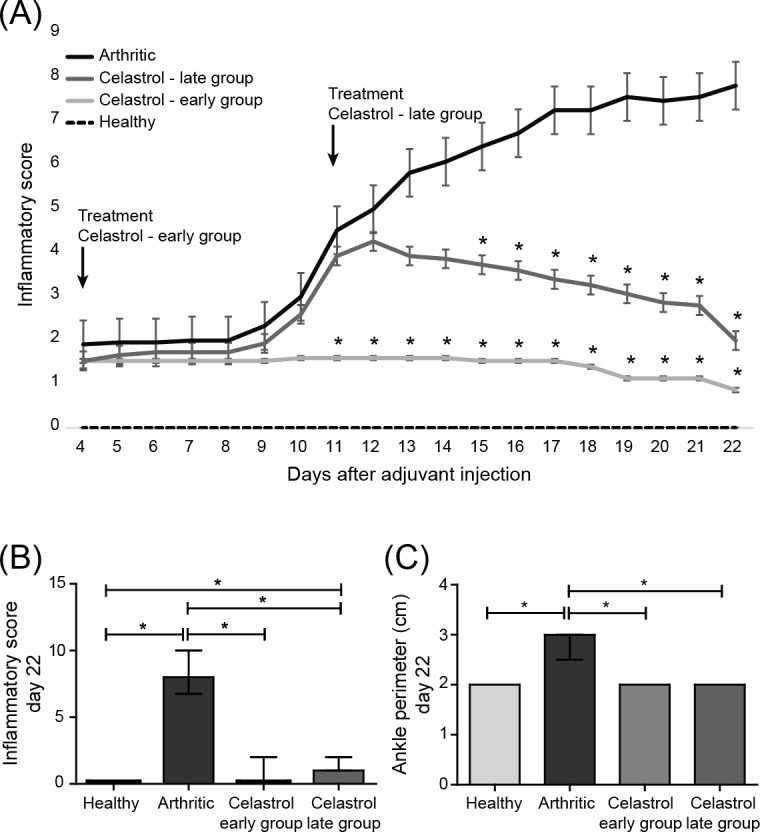
(A) Celastrol ameliorates inflammation throughout time. Notice that after 7 days of treatment celastrol early-treated rats presented minimal inflammatory activity, whereas arthritic rats started to increase the inflammatory manifestations sharply. Arrows indicate the beginning of treatment after 4 and 11 days of disease induction. (B) Celastrol improves the clinical outcome in adjuvant-induced arthritic rats. Inflammatory score in celastrol-treated AIA rats is maintained significantly diminished in comparison with arthritic rats. (C) Celastrol suppresses the progression of swelling in the left hind paw. Left paw edema/swelling is markedly present in arthritic rats in contrast to celastrol-treated animals. Data are expressed as median with interquartile range. Differences were considered statistically significant for p-values<0.05, according to the Kruskal-Wallis (Dunn´s Multiple Comparison tests) and Mann–Whitney tests. Healthy N = 19, Arthritic N = 23, Celastrol early group N = 15 and Celastrol late group N = 15.

Celastrol was intraperitoneally administrated at a dose of 1μg/g/daily after 4 days of disease induction (early treatment group) and after 11 days of disease induction (late treatment group) [17]. The inflammatory score and ankle perimeter were evaluated during the treatment period (Fig 1 and S1 Fig). As shown in Fig 1A, all animals already presented signs of arthritis by the fourth day of disease induction and after 9 days the untreated arthritic group started to increase the inflammatory manifestations sharply. In contrast, in early celastrol-treated rats there was minimal inflammatory activity or even complete abrogation of arthritis manifestations. In the late treatment group, drug administration was started when animals already presented a mean inflammatory score of 4, but celastrol still caused a significant decrease of arthritis manifestations over time. In fact, the only remaining sign of swelling was observed in most animals in the local of injection of the adjuvant, for disease induction. This result shows that this drug has a significant anti-inflammatory effect even when administrated at a later phase of arthritis development. Celastrol showed a significant anti-inflammatory effect, as assessed by the evaluation of the inflammatory score (p<0.0001 in early and late treatment groups vs. arthritic animals, shown in Fig 1B) and also by the measurement of ankle perimeter (p<0.0001 in early and late treatment groups vs. arthritic animals, shown in Fig 1C). Of note, by the end of the treatment, at day 22, there were no significant differences between the celastrol early and late treatment groups. Importantly, both treated groups showed a significant reduction in the inflammatory score when compared with their baselines (p = 0.0002 in celastrol early-treated vs. arthritic rats sacrificed at day 4 and p<0.0001 in celastrol-late treated vs. arthritic rats sacrificed at day 11).

Up to now significant adverse effects of celastrol administration have not been reported. However the few toxicological analysis of this compound *in vivo* were based in data from the assessment of animal mortality and some blood parameters in studies using Tripterygium wilfordii plant extracts [49]. To investigate the potential side effects of pure celastrol administration in AIA rats, we performed liver and renal function tests, such as the measurement of creatine kinase, urea, lactate dehydrogenase and alanine transaminase in serum samples collected at the time of sacrifice. No significant differences were observed in these parameters when comparing arthritic rats with animals under treatment (p = 0.2). In addition, a pathologist blinded to experimental groups examined the tissue histological sections and has reported no evidence of drug-induced liver or renal injury, as well as no lung or spleen alterations (S2 Fig). Of note, body weight variations were recorded throughout treatment duration, and no weight loss was observed due to celastrol administration (p = 0.1265 and p = 0.6005 in celastrol early and late treatment groups vs. arthritic rats, respectively). Contrarily, there was an association between disease activity and weigh loss (p = 0.0273 in arthritic rats vs. healthy animals). In fact, in the late treatment group, animals started to lose weight due to disease activity and after treatment was initiated no more weight loss was observed (p = 0.0436 in late-treated rats at day 11 vs. day 4, and p = 0.9009 in late-treated rats at day 22 vs. day 11) (S3 Fig). Importantly, administration of celastrol has already been tested in healthy animals in a wide range of concentrations [21]. So far, there are no data showing deleterious effects at a dose of 1mg/kg (the concentration used in this work).

### Celastrol diminishes systemic proinflammatory cytokine IL-6 *in vivo*

**Fig 2:**
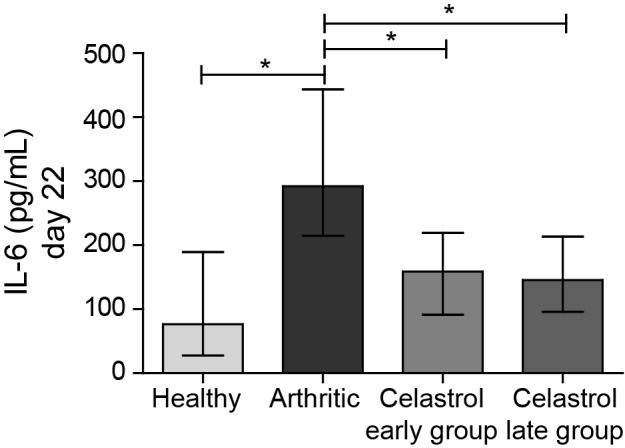
Celastrol reduces the serum levels of IL-6 in arthritic rats. Notice that celastrol treatment significantly reduces the systemic concentration of the proinflammatory cytokine IL-6 to levels similar to healthy controls. Data are expressed as median with interquartile range. Differences were considered statistically significant for p-values<0.05, according to the Kruskal-Wallis (Dunn´s Multiple Comparison tests) and Mann–Whitney tests. Healthy N = 21, Arthritic N = 23, Celastrol early group N = 15 and Celastrol late group N = 15.

Proinflammatory cytokines, namely IL-1β, IL-6, IL-17 and TNF act synergistically to maintain inflammation and bone erosions in animal models of arthritis and in RA patients. These cytokines activate the NF-kB pathway that in turn leads to the downstream up-regulation of several cytokines, chemokines and MMPs, which are responsible for the inflammatory process and for the destruction of cartilage and bone. We therefore aimed at evaluating the anti-inflammatory effect of celastrol on the peripheral circulating levels of these cytokines. We have observed that IL-6 levels increase in the serum of AIA rats throughout the course of arthritis, although abundant production was seen only after 2 weeks of disease onset. Thus, IL-6, which is produced by monocytes/macrophages, T cells and synovial fibroblasts [50], seems to be involved in the systemic events underlying arthritis, especially in the transition phase of its development. Fig 2 shows that celastrol administration significantly reduces the levels of IL-6 detected in peripheral blood, both in early and late treatment groups (p<0.0001 in both groups vs. arthritic rats after 22 days of disease induction), presenting a cytokine concentration similar to healthy controls. Importantly, both treated groups showed a significant reduction in the circulating levels of IL-6 when compared with their baselines (p = 0.0387 in celastrol early-treated vs. arthritic rats sacrificed at day 4 and p<0.0001 in celastrol-late treated vs. arthritic rats sacrificed at day 11). This observation is corroborated by data already published which shows that IL-6 mRNA is decreased after celastrol treatment *in vitro* [51]. We have also quantified the circulating concentration of IL-1β, IL-17 and TNF, but no differences were found when comparing arthritic rats with animals under celastrol treatment or with healthy controls (p>0.05, S4 Fig), possibly because these cytokines are not increased in the periphery at this stage of disease development. Previously, we have demonstrated that circulating IL-1β and IL-17 are only increased in the early phase of RA, in contrast to IL-6, which was found to be increased also in the later phase of the disease [18], arguing that the detection of these cytokines in the periphery is dependent on disease evolution. In addition, literature controversy highlights the likelihood that systemic markers and mediators of arthritis might not fully reflect the underlying local disease progression. AIA rat model have increased levels of IL-1β (since the preclinical disease stage), IL-6, IL-17 and TNF (in the acute and chronic stages) locally in the joints [42]. Recently, it has been shown in the same animal model that both Tripterygium and celastrol decrease the levels of these cytokines locally in the arthritic joints [19, 20, 22, 52].

### Celastrol ameliorates local joint inflammation and bone damage in AIA rats

**Fig 3:**
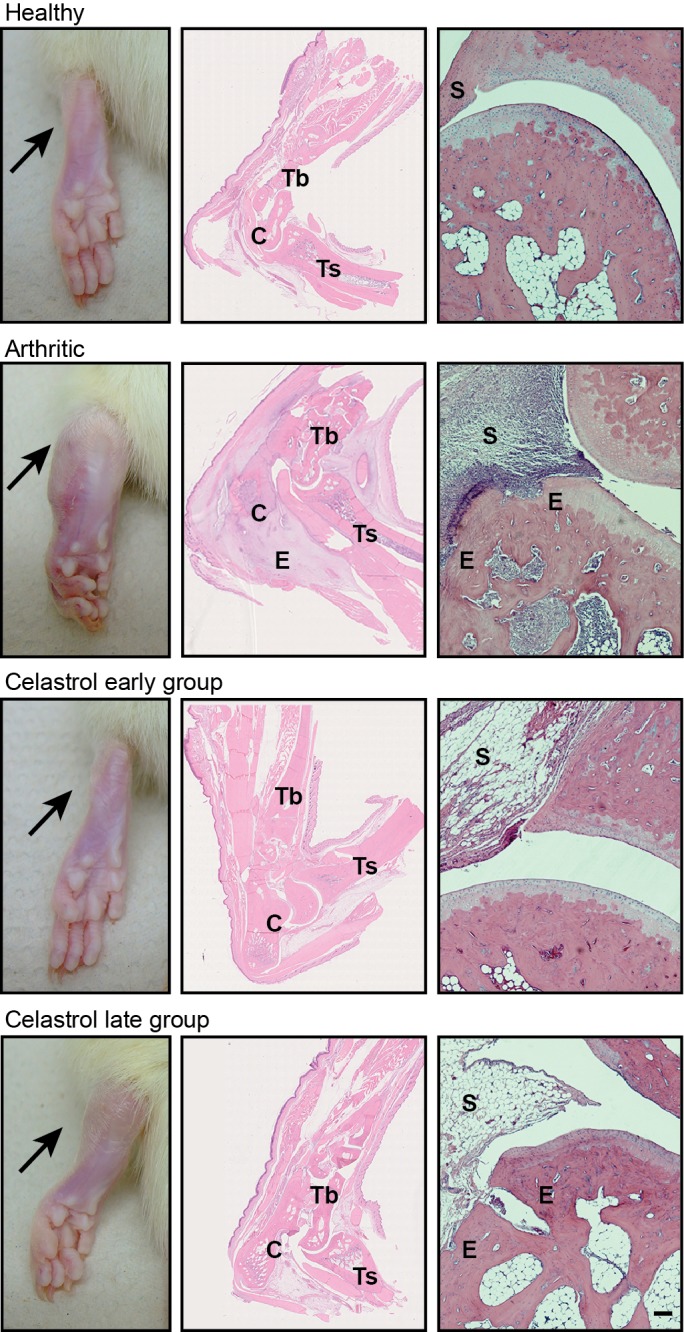
Histological images of joints after celastrol treatment. These patterns are merely illustrative of the type of histological features observed. Black arrow indicates the absence/presence of ankle swelling in rat hind paws. C–calcaneus, E–edema or erosion, S–synovia, Tb–tibia, Ts–tarso. Magnification of 50×. Bar: 100 μm.

**Fig 4:**
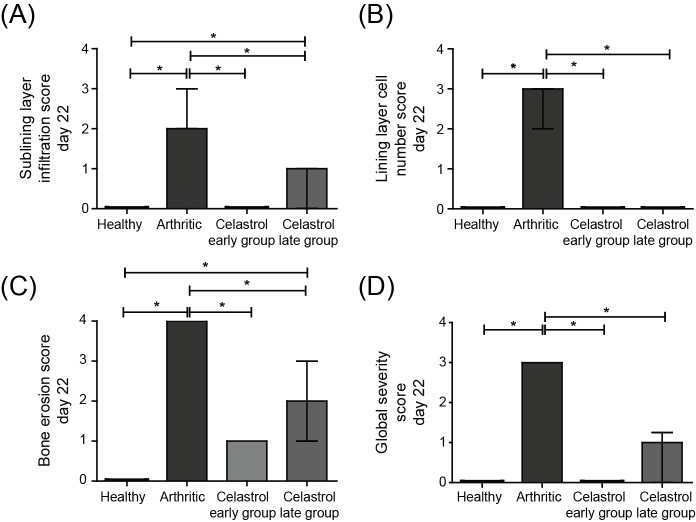
Celastrol suppresses arthritic inflammation and tissue damage locally in the joints of AIA rats. A semi-quantitative evaluation of histological sections was performed. Notice that celastrol has inhibited cellular infiltration (A), completely reversed the number of lining layer cells to the normal values (B) and prevented bone erosion occurrence (C), allowing for a normal joint structure comparable to healthy rats in both early and late treatment groups (D). Data are expressed as median with interquartile range. Differences were considered statistically significant for p-values<0.05, according to the Kruskal-Wallis (Dunn´s Multiple Comparison tests) and Mann–Whitney tests. Correlation analysis was performed using the Spearman test. Healthy N = 19, Arthritic N = 23, Celastrol early group N = 15 and Celastrol late group N = 15.

To evaluate the effect of celastrol in the preservation of local articular joint synovium and bone structures, paw sections stained with hematoxylin and eosin were performed (illustrative images can be observed in Fig 3). The histological evaluation using 4 semi-quantitative scores is depicted in Fig 4.

The levels of the sublining layer infiltration (Fig 4A) and the lining layer cell numbers (Fig 4B) started to augment immediately after 4 days of disease onset and continued to markedly increase until the end of the study (p<0.0001, healthy vs. arthritic rats sacrificed after 22 days of disease induction). The data from Fig 4D revealed that rats treated with celastrol had a normal joint structure at the end of the study period, with an abrogation of the inflammatory infiltrate and a reduction of the number of cells present in the lining layer of the synovial membrane (p<0.0001 in early and late treatment groups vs. arthritic animals). Moreover, when comparing the infiltration score of celastrol early-treated group with diseased animals at baseline (day 4), we observed that there was a complete clearance of the cellular infiltrate (p = 0.0006 in the early-treated group sacrificed at the end of the treatment period vs. arthritic rats sacrificed at baseline of the treatment period, i.e. after 4 days of disease induction), with a phenotype similar to a healthy control. Regarding the analysis of the lining layer cell number score (Fig 4B), data showed that both celastrol early and late treatment groups have dramatically reduced scores, in comparison with the animals at the beginning of treatment, corresponding to baseline (p = 0.0107 in early-treated arthritic rats sacrificed at the end of the study period vs. arthritic rats sacrificed at baseline, at day 4 and p<0.0001 in late-treated arthritic rats sacrificed at the end of the study period vs. arthritic rats sacrificed at baseline, at day 11, respectively).

Celastrol is also effective in preventing bone articular destruction as shown in Fig 4C. The development of bone erosions in the AIA rat model occurred immediately after 4 days of disease onset, and markedly increased throughout the development of arthritis (p<0.0001 in healthy vs. arthritic rats sacrificed after 22 days of disease induction), with a strong correlation between erosion and infiltration as well as with proliferation scores (r^2^ = 0.70, p = 0.0009 and r^2^ = 0.97, p<0.0001, respectively). By the end of the treatment course, celastrol was able to suppress the appearance of bone erosions (p<0.0001 in both celastrol early and late treatment groups vs. arthritic rats), maintaining the phenotype similar to their baselines. These results might suggest that celastrol is able to modulate oscleoclast pathways. In fact, a study has demonstrated that celastrol inhibits the formation and activity of mature osteoclasts, induces their apoptosis and reduces osteoblast viability and activity *in vitro* [53].

Overall, these data are supported by studies already published in the literature using several plant extracts and different experimental outlines [19, 20, 22, 54, 55]. Thus, there is strong evidence that celastrol is able to significantly diminish inflammation and bone damage, even when administrated in a later phase of arthritis development.

### Celastrol inhibits synovial lymphocyte infiltration and cell proliferation in arthritic rat joints

**Fig 5:**
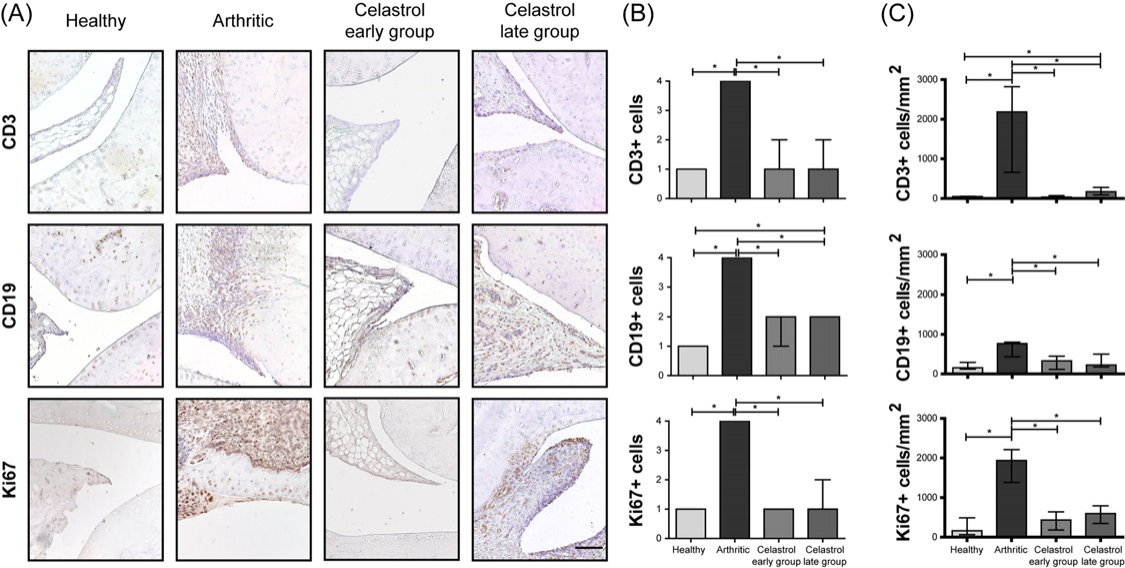
Celastrol reduces the number of T cells and B cells present in the synovial membrane, and suppresses synovial cell proliferation. (A) Representation of the immunohistochemical evaluation performed in paw sections at day 22 after celastrol treatment. Magnifications of 200×. Bar: 100 μm. (B) Immunohistochemical analysis was performed using a semi-quantitative score. Notice that both celastrol early and late-treated rats showed a significant reduction in the number of CD3 and CD19 positive cells as well as a reduction in the levels of synovial cell proliferation assessed by Ki67 marker in comparison with arthritic rats at day 22. Healthy N = 16, Arthritic N = 10, Celastrol early group N = 15 and Celastrol late group N = 15. (C) Immunohistochemical quantification was performed using an image analysis software written in MATLAB to identify and count the number of positive cells for each antibody in representative sections. Notice that both celastrol early and late-treated rats showed a significant reduction in the number of CD3, CD19 and Ki67 positive cells in comparison with arthritic rats at day 22. Healthy N = 5, Arthritic N = 5, Celastrol early group N = 5 and Celastrol late group N = 5. Data are expressed as median with interquartile range. Differences were considered statistically significant for p-values<0.05, according to the Kruskal-Wallis (Dunn´s Multiple Comparison tests) and Mann–Whitney tests.

The immunohistochemical analysis revealed that arthritic rats treated with celastrol have reduced levels of lymphocyte infiltration into the joints (Fig 5). As can be observed in Fig 5B there were significant reductions of CD3+ T cells (p<0.0001 in early and late treatment groups vs. arthritic rats) and CD19+ B cells (p<0.0001 in early and late treatment groups vs. arthritic rats). In contrast, the number of these cells markedly increased throughout disease progression in untreated animals (p<0.0001 in healthy vs. arthritic rats, sacrificed at the end of the study period). A study by Venkatesha et al, have shown that celastrol reduces the level of chemokines, which might explain the inhibition of leukocyte migration [20].

In addition, we have also studied cell proliferation by staining joint tissue sections with the Ki67 marker. The immunohistochemical results shown in Fig 5B revealed that animals treated with celastrol have reduced levels of synovial cell proliferation in both early and late treated rats (p<0.0001 in both groups vs. arthritic animals), with a score similar to the healthy controls.

Results of immunohistochemical quantification also showed that celastrol significantly reduced CD3+ T cells (p = 0.0079 in both early and late treatment groups vs. arthritic rats) and CD19+ B cells (p = 0.0317 in both early and late treatment groups vs. arthritic rats) infiltrated into the joints as well as synovial cell proliferation (p = 0.0079 in both early and late treatment groups vs. arthritic rats), as depicted in Fig 5C.

### Celastrol significantly reduces CD68+ macrophages in the arthritic synovial tissue

**Fig 6:**
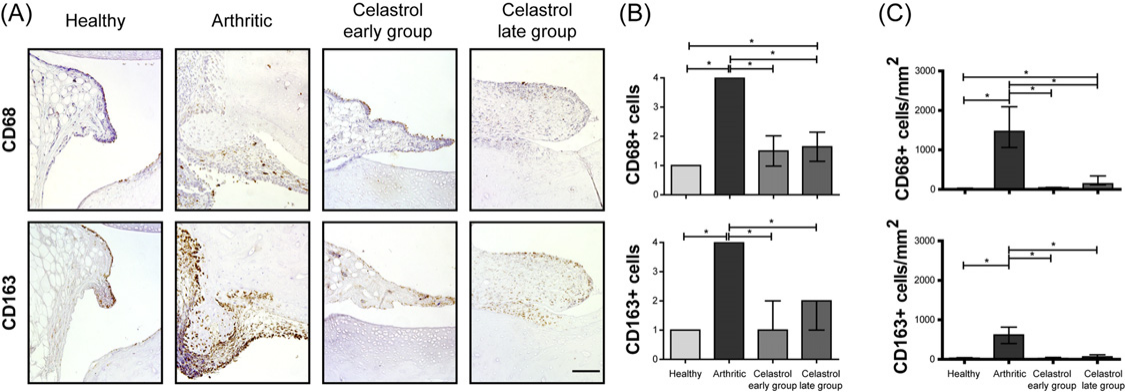
Celastrol reduces the number of synovial CD68+ macrophages. (A) Representation of the immunohistochemical evaluation performed in paw sections at day 22 after celastrol treatment. Magnifications of 200×. Bar: 100 μm. (B) Immunohistochemical analysis was performed using a semi-quantitative score. Notice that both celastrol early and late-treated rats showed a significant reduction in the number of CD68 and CD163 positive cells in comparison with arthritic rats at day 22. Healthy N = 16, Arthritic N = 10, Celastrol early group N = 15 and Celastrol late group N = 15. (C) Immunohistochemical quantification was performed using an image analysis software written in MATLAB to identify and count the number of positive cells for each antibody in representative sections. Notice that both celastrol early and late-treated rats showed a significant reduction in the number of CD68 and CD163 positive cells in comparison with arthritic rats at day 22. Healthy N = 5, Arthritic N = 5, Celastrol early group N = 5 and Celastrol late group N = 5. Data are expressed as median with interquartile range. Differences were considered statistically significant for p-values<0.05, according to the Kruskal-Wallis (Dunn´s Multiple Comparison tests) and Mann–Whitney tests.

The activated macrophages in the synovium are derived from circulating monocytes and secrete various mediators that participate in arthritis induction and tissue injury. Studies of drug efficacy in RA patients have identified, from a large panel of synovial biomarkers, sublining CD68+ macrophages as an optimal marker to evaluate clinical response, with an association between clinical improvement and the reduction of CD68+ macrophage scores. Therefore, CD68+ sublining macrophages have been recognized as a synovial biomarker, with a high sensitivity in discriminating between effective and ineffective therapies or placebo, useful in an early stage of drug development [34, 56]. We have thus performed the characterization of CD68+ macrophages present in the synovial tissue after treatment with celastrol (Fig 6). Arthritic rats have shown an increase in the number of CD68+ synovial macrophages throughout the development of the disease (p<0.0001 in healthy vs. arthritic rats, as shown in Fig 6B). Importantly, celastrol significantly decreased the number of CD68+ macrophages infiltrated into the arthritic joint tissue (p<0.0001 in early and late treatment groups vs. arthritic rats). In addition, celastrol administration significantly decreased the levels of CD163+ macrophages (p<0.0001 in early and late treatment groups vs. arthritic rats). CD163 is a useful marker in this context because it is a more selective macrophage marker and helps to discriminate between synovial macrophages and synovial intimal fibroblasts, which also stain positively for CD68 in RA synovium [57]. Previous studies have in fact shown that synovial intimal fibroblasts migration and invasion into the synovium are also reduced by celastrol [55, 58].

Results of immunohistochemical quantification shown in Fig 6C also revealed that celastrol significantly reduced CD68+ cells (p = 0.0079 in both early and late treatment groups vs. arthritic rats) and CD163+ macrophages (p = 0.0079 in both early and late treatment groups vs. arthritic rats) infiltrated into the joints.

Because inflammatory synovial tissue macrophages are derived from peripheral blood monocytes, these observations suggest decreased monocyte recruitment into the joints of arthritic rats treated with celastrol, even when treatment was initiated in a later phase of disease development.

## Discussion

In this study, we have shown that celastrol substantially depletes CD68+ sublining synovial cells, considered to be the biomarker with the strongest association with response to treatment in RA. Moreover, celastrol was effective and safe in suppressing synovial inflammation and bone damage in rats with AIA.

We have consistently observed that celastrol treatment reduced serum IL-6 levels in arthritic rats. This observation is relevant because IL-6 is a proinflammatory cytokine that plays a relevant role in the pathogenesis of RA, namely in Th17 polarization and plasma B cell differentiation, in the production of chemokines, adhesion molecules, and VEGF, and in the secretion of RANKL and MMPs, amplifying inflammatory cell infiltration and inducing osteoclastogenesis [59–61]. Interestingly, it was shown that celastrol can suppress arthritis in part by altering Th17/Treg ratio in inflamed joints [52]. Additionally, celastrol-treated rats showed a significant reduction in the severity of clinical arthritis as well as in pannus formation and leukocyte cell infiltration into the joint synovial tissue. This cell infiltration and proliferation inhibitory effect of celastrol may thus prove to be of interest to prevent and treat the development of the synovial tumor-like pannus tissue characteristic of established RA and responsible for bone damage. Interestingly, histological analysis also revealed that celastrol is effective in suppressing local inflammation-induced bone loss. Of note, celastrol treatment is effective when administrated both in the early and established phase of arthritis, which is relevant for the potential clinical implications of our findings. Our report is the first to demonstrate the protective coupled effect of celastrol *in vivo* on both synovial inflammation and joint bone damage restoring synovial homeostasis, fulfilling this unmet medical need in RA treatment approach. Importantly, CD68+ sublining macrophages, a synovial biomarker with a high sensitivity in selecting effective RA therapies in an early stage of drug development, is significantly reduced in the synovia of celastrol-treated rats.

It has already been reported that celastrol targets NF-kB, via long-lasting inhibition of IKKβ activity [62]. In fact, the inactivation of NF-kB in animal models has shown the ability to suppress arthritis [63]. NF-kB participates in the transcription of genes encoding many proinflammatory cytokines and chemokines, in the regulation of different immune cells and in the expression of adhesion molecules and matrix MMPs [64]. Based on microarray gene expression profile it has been demonstrated that celastrol represses cell proliferation, inflammation and immune responses (targets T and B cells, antigen processing and presentation), blocks metabolic pathways, has anti-oxidant properties, and targets VEGF, proinflammatory cytokines and chemokines [65]. Indeed, it has been demonstrated that celastrol reduces the levels of chemokines, possibly affecting leukocyte migration [20]. Celastrol has thus a broad spectrum of targets, modulating immune responses rather than inducing immunosuppression [65]. Our results point out that pure celastrol used in the AIA rat model is not associated with increased risk of infections, have no hepatotoxicity or nephrotoxicity, suggesting that at least for short-term RA treatment, celastrol might be a safe drug.

Overall, our results validate celastrol as a promising compound for the treatment of inflammation and inflammation-induced bone damage and provide relevant insights into the usage of celastrol as a future drug for RA. It would be interesting to extend this knowledge by studying the anti-arthritic properties of celastrol *in vivo* using different animal models of arthritis, namely the CIA model, and evaluate differences in efficacy depending on animal gender.

## Supporting Information

S1 Fig Ankle perimeter kinetics. Celastrol was administered to AIA rats both in the early (4 days after disease induction) and late (11 days after disease induction) phases of arthritis development. Notice that after 7 days of treatment celastrol early-treated rats presented an ankle perimeter similar to the healthy control, whereas arthritic rats started to increase left ankle edema/swelling sharply. In the celastrol late-treated group, ankle swelling started to increase in parallel to the augment of the inflammatory score, but after treatment was initiated ankle perimeter started to significantly decrease. Data are expressed as median with interquartile range. Differences were considered statistically significant for p-values<0.05, according to the Kruskal-Wallis (Dunn´s Multiple Comparison tests) and Mann–Whitney tests. Healthy N = 19, Arthritic N = 23, Celastrol early group N = 15 and Celastrol late group N = 15.
(TIF)

S2 Fig Administration of pure celastrol induces no hepatic or renal toxicity. At day 22 after disease induction no hepatocellular or renal lesion was observed in any of the animals. Liver and kidney samples from all animals were analyzed by a pathologist blinded to experimental groups but only representative histological sections are shown. H&E staining; Magnifications of 100×. Bar: 300 μm.
(TIF)

S3 Fig Celastrol treatment has no effect on body weight. Notice that no weight loss was observed due to celastrol administration. In contrast, there was an association between disease activity and weight loss, which was highlighted in late-treated rats that started to lose weight due to disease activity (day 4 up to day 11) and after treatment was initiated no more weight loss was observed (day 11 up to day 22). Data are expressed as median with interquartile range. Differences were considered statistically significant for p-values<0.05, according to the Mann–Whitney tests.
(TIF)

S4 Fig Celastrol has no effect in the serum levels of IL-1β, IL-17 and TNF in arthritic rats. Data are expressed as median with interquartile range. Differences were considered statistically significant for p-values<0.05, according to the Kruskal-Wallis (Dunn´s Multiple Comparison tests) and Mann–Whitney tests. Healthy N = 19, Arthritic N = 23, Celastrol early group N = 15 and Celastrol late group N = 15.
(TIF)
