# Supplementary figures and images for: Decrease of CD68 Synovial Macrophages in Celastrol Treated Arthritic Rats
Source: PLoS One. 2015 Dec 11;10(12):e0142448. doi: 10.1371/journal.pone.0142448 (PMC4676706; doi:10.1371/journal.pone.0142448)

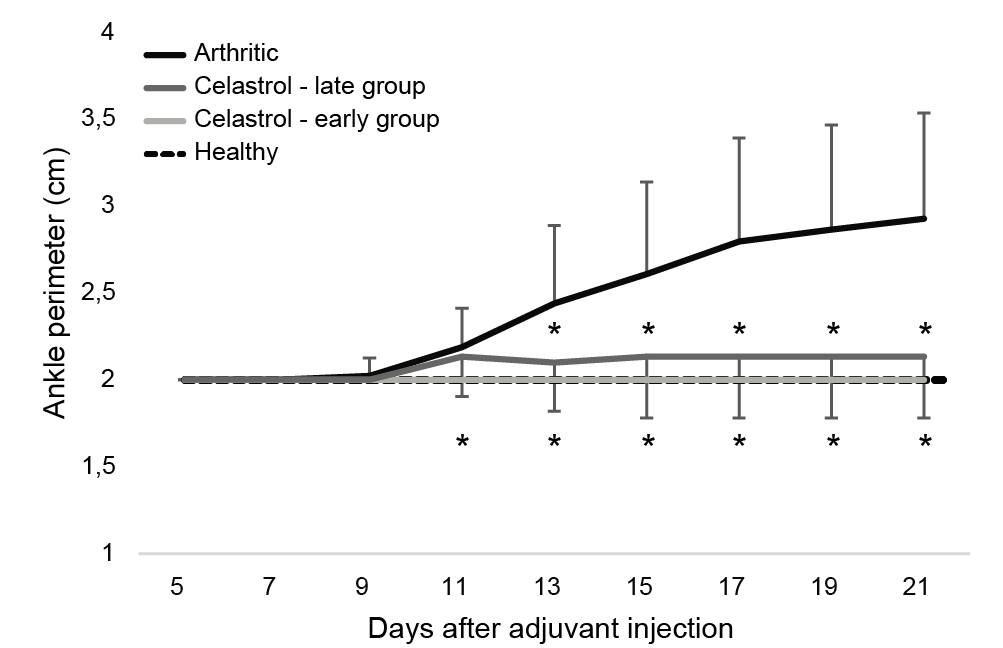

Supplement: S1 Fig — Celastrol was administered to AIA rats both in the early (4 days after disease induction) and late (11 days after disease induction) phases of arthritis development. Notice that after 7 days of treatment celastrol early-treated rats presented an ankle perimeter similar to the healthy control, whereas arthritic rats started to increase left ankle edema/swelling sharply. In the celastrol late-treated group, ankle swelling started to increase in parallel to the augment of the inflammatory score, but after treatment was initiated ankle perimeter started to significantly decrease. Data are expressed as median with interquartile range. Differences were considered statistically significant for p-values<0.05, according to the Kruskal-Wallis (Dunn´s Multiple Comparison tests) and Mann–Whitney tests. Healthy N = 19, Arthritic N = 23, Celastrol early group N = 15 and Celastrol late group N = 15. (TIF) [file pone.0142448.s001.tif]

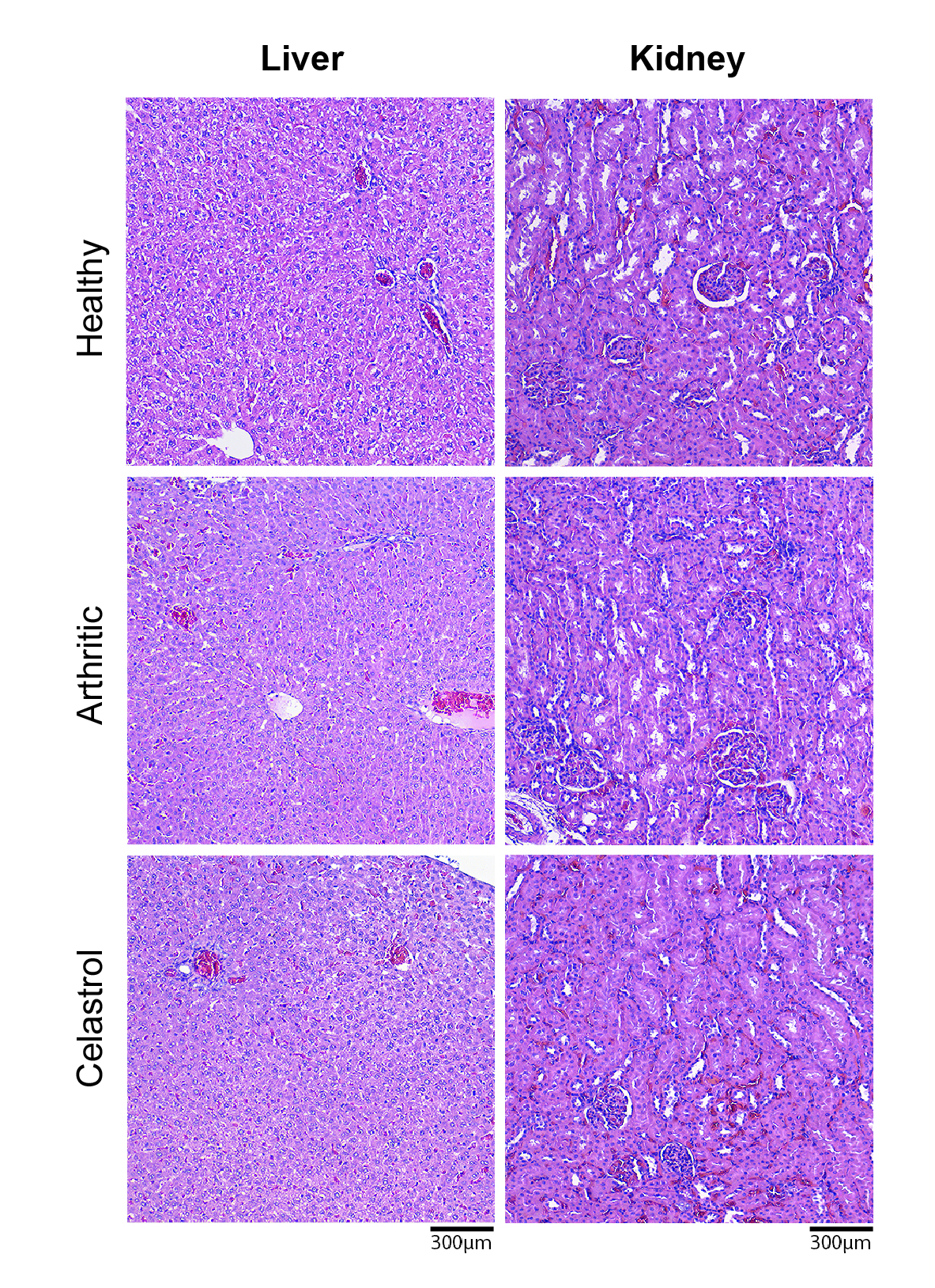

Supplement: S2 Fig — At day 22 after disease induction no hepatocellular or renal lesion was observed in any of the animals. Liver and kidney samples from all animals were analyzed by a pathologist blinded to experimental groups but only representative histological sections are shown. H&E staining; Magnifications of 100×. Bar: 300 μm. (TIF) [file pone.0142448.s002.tif]

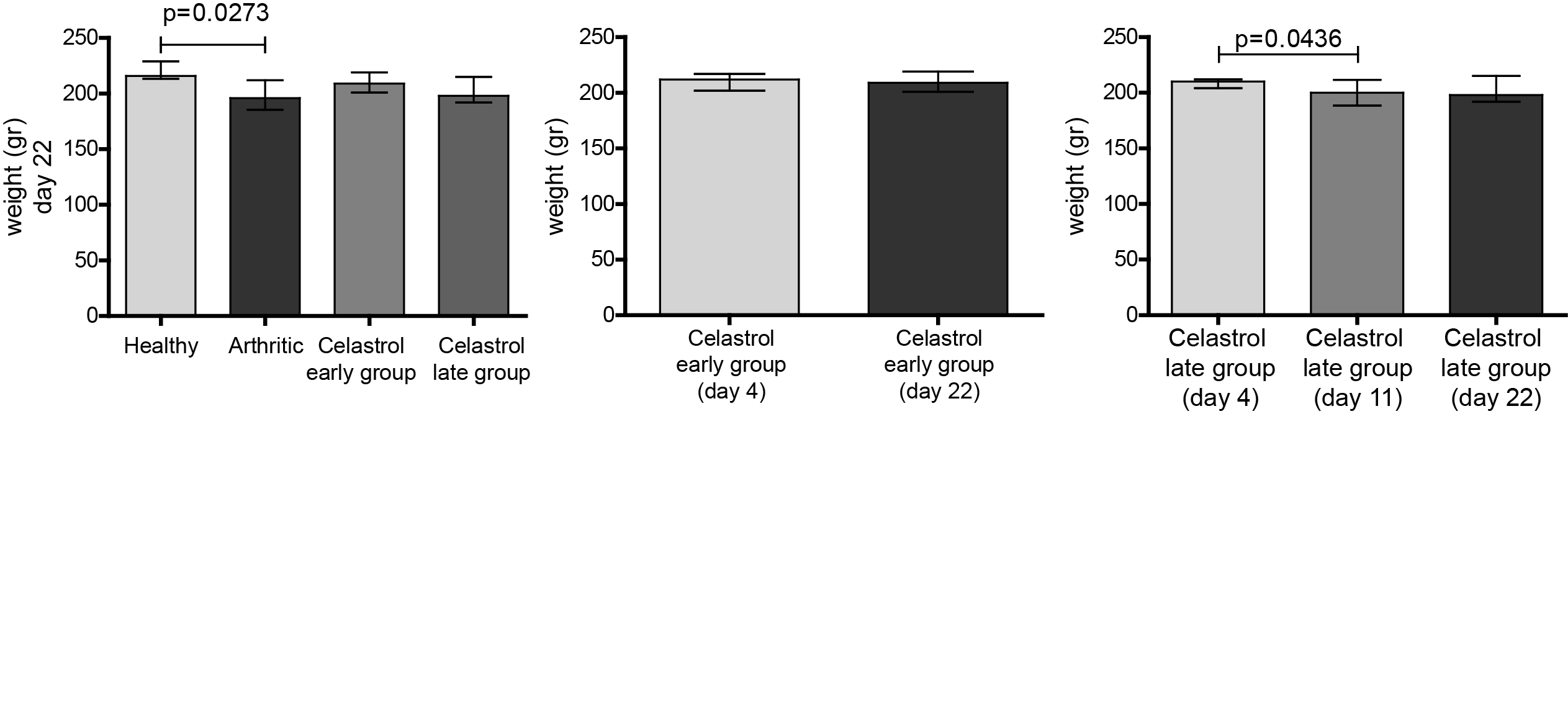

Supplement: S3 Fig — Notice that no weight loss was observed due to celastrol administration. In contrast, there was an association between disease activity and weight loss, which was highlighted in late-treated rats that started to lose weight due to disease activity (day 4 up to day 11) and after treatment was initiated no more weight loss was observed (day 11 up to day 22). Data are expressed as median with interquartile range. Differences were considered statistically significant for p-values<0.05, according to the Mann–Whitney tests. (TIF) [file pone.0142448.s003.tif]

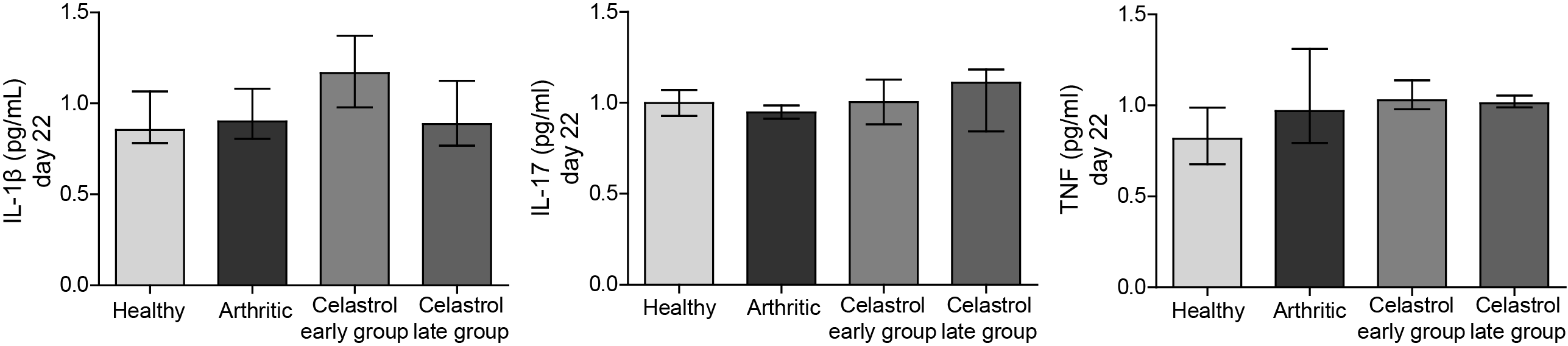

Supplement: S4 Fig — Data are expressed as median with interquartile range. Differences were considered statistically significant for p-values<0.05, according to the Kruskal-Wallis (Dunn´s Multiple Comparison tests) and Mann–Whitney tests. Healthy N = 19, Arthritic N = 23, Celastrol early group N = 15 and Celastrol late group N = 15. (TIF) [file pone.0142448.s004.tif]
